# Hybrid pointer networks for traveling salesman problems optimization

**DOI:** 10.1371/journal.pone.0260995

**Published:** 2021-12-14

**Authors:** Ahmed Stohy, Heba-Tullah Abdelhakam, Sayed Ali, Mohammed Elhenawy, Abdallah A. Hassan, Mahmoud Masoud, Sebastien Glaser, Andry Rakotonirainy

**Affiliations:** 1 Department of Computer and Systems Engineering, Minya University, Minya, Egypt; 2 Centre for Accident Research and Road Safety, Queensland University of Technology, Brisbane, Australia; Torrens University Australia, AUSTRALIA

## Abstract

In this work, we proposed a hybrid pointer network (HPN), an end-to-end deep reinforcement learning architecture is provided to tackle the travelling salesman problem (TSP). HPN builds upon graph pointer networks, an extension of pointer networks with an additional graph embedding layer. HPN combines the graph embedding layer with the transformer’s encoder to produce multiple embeddings for the feature context. We conducted extensive experimental work to compare HPN and Graph pointer network (GPN). For the sack of fairness, we used the same setting as proposed in GPN paper. The experimental results show that our network significantly outperforms the original graph pointer network for small and large-scale problems. For example, it reduced the cost for travelling salesman problems with 50 cities/nodes (TSP50) from 5.959 to 5.706 without utilizing 2opt. Moreover, we solved benchmark instances of variable sizes using HPN and GPN. The cost of the solutions and the testing times are compared using Linear mixed effect models. We found that our model yields statistically significant better solutions in terms of the total trip cost. We make our data, models, and code publicly available https://github.com/AhmedStohy/Hybrid-Pointer-Networks.

## I. Introduction

Combinatorial optimization problems have garnered substantial attention from the theory and algorithm design community in recent decades as fundamental challenges in computer science and operations research. TSP in one fundamental combinatorial optimization problem that have been explored in the disciplines of logistics transportation, genomics, express delivery, and dispatching. TSP is often defined on a graph with a number of nodes, and it is essential to search through the permutation sequences of nodes for finding an optimal one with the shortest traveling distance.

Due to the many applications of the travelling salesman problem (TSP) in many areas, it has received significant attention from the machine learning community in the past years. However, the developed neural combinatorial optimization models are still in the infantry stage. Generalization is still an unresolved problem when it comes to dealing with many points with high precision. The travelling salesman problem (TSP) is considered as one of the most significant and practical problems. Consider a salesman travelling to several areas; the salesman must visit each city just once while minimizing the total travel time. TSP is an NP-hard problem [1], which addresses the challenge of finding the optimal solution in polynomial time.

Exact algorithms, approximation algorithms, and heuristic algorithms are examples of traditional approaches for tackling NP-hard graph optimization problems [2]. Exact algorithms using the branch and bound framework can produce optimum solutions, but due to their NP-hardness, they are not suited for large-scale applications. Polynomial-time approximation algorithms can often produce quality-guaranteed solutions, although they have lower optimality guarantees than precise algorithms. The optimality guarantee may not exist at all for situations that are not amenable to a polynomial approximation approach. Furthermore, because of their high computing efficiency, heuristic algorithms are commonly employed, although they typically need adaptations and subject specialist understanding for a given situation. Heuristic algorithms frequently lack theoretical basis, all three groups of algorithms previously mentioned seldom take advantage of the common features among optimization problems, and thus frequently require the design of a new algorithm to solve a different instance of an even similar problem that is based on the same combinatorial structure, with the coefficient values in the objective function or constraints regarded as samples from the same basic distributions [3]. The use of machine learning methodologies has provided a silver lining in the form of a scalable solution for solving combinatorial problems with similar combinatorial structures.

Many approximation algorithms and heuristics, such as Christofides algorithm [4], local search [5], and the Lin-Kernighan heuristic (LKH) [6] have been developed to overcome the complexity of the exact algorithms which are guaranteed to yield an optimal solution but are frequently too computationally costly to be utilized in practice [7]. Many combinatorial optimization problems, such as a TSP has a graph structure [8], which may be easily described using the current graph embedding or network embedding techniques. The graph information is integrated in a continuous node representation in this method. Because of its great skills in information embedding and belief propagation of graph topology, the most recent development of graph neural network (GNN) may be applied in simulating a graph combinatorial problem [9]. This drives us to use a GNN model to handle combinatorial optimization problems, specifically TSP.

The pointer network [10], a seq2seq model [11], shows great potential for approximation solutions to combinatorial optimization problems such as identifying the convex hull and the TSP. It uses LSTM [12] as the encoder and an attention mechanism [13] as the decoder to extract features from city coordinates. It then predicts a policy outlining the next likely city by selecting a permutation of visited cities. The pointer network model is trained using the Actor-Critic technique [14].

Moreover, the attention model [15, 16], influenced by the Transformer architecture [13], tried to address routing problems such as the TSP and VRP. Graph pointer networks [17] extended the traditional pointer networks with an additional layer of graph embedding, this transformation achieved a better generalization for a large-scale problem, but the GPN model without 2-opt still struggling for finding the optimal solutions for small and large scale.

GPN has limited capabilities for tackling small-scale problems, and the suggested GNN employed in its architecture isn’t the ideal encoder for determining point-to-point relationships. The work proposed in this paper begins with how the performance of graph pointer networks can be improved without changing much of the architecture; an extra encoder layer is added alongside the graph embedding layer to act as a hybrid encoder, and this gives the model the ability to achieve good results; this will be discussed in greater detail in the HPN section.

Extensive experimental work results show that the proposed technique significantly outperforms previous DL-based methods on TSP. The learnt model is more successful than standard hand-crafted rules in guiding the improvement process, and they may be further strengthened by simple ensemble methods. Furthermore, HPN generalizes rather well to a variety of problem sizes, starting solutions, and even real-world datasets. It should be noted that the goal is not to outperform highly optimized and specialized traditional solvers but to present a generalized model that can automatically learn good search heuristics on different problem sizes, which has a great value when applied to real-world problems.

In this work, we propose a deep reinforcement learning model trained via Actor-critic. Our architecture is based on a pointer attention mechanism that outputs nodes sequentially for action selection. We introduce a reinforcement learning formulation to learn a stochastic policy of the next promising solutions using a hybrid architecture, incorporating the search’s history information by keeping track of the current best-visited solution. Our results show that we can learn policies for the Euclidean TSP that achieve the state-of-the-art solution compared with previous work in RL-based Models. Moreover, our approach can achieve state of the art results compared with previous deep learning methods based on construction [10, 15, 18–20] and improvement [21] heuristics.

## II. Travelling salesman problem (TSP)

TSP is a classic example of a combinatorial optimization problem that has been used in data clustering, genome sequencing, as well as other fields. TSP problem is NP-hard, and several exact, heuristic and approximation algorithms have been developed to solve it. In this paper, TSP problems are assumed to be symmetric. The symmetric TSP is regarded as an undirected graph.

### 1. Asymmetric vs. symmetric TSP

The distance between two cities in the symmetric TSP is the same in each opposite direction, producing an undirected network. This symmetry cuts the number of alternative solutions in half. Paths may not exist in both directions in the asymmetric TSP, or the distances may be different, resulting in a directed graph.

### 2. Directed vs. undirected graphs

Edges in undirected graphs do not have a direction. Each edge may be travelled in both directions, indicating a two-way connection. The edges of directed graphs have a direction. The edges represent a one-way connection, as each edge may only be travelled in one direction.

A full undirected graph can be defined as *C* = (*V*,*E*)where V is the vector of vertices of graph C, and E is the vector of edges between these vertices. In this study, the TSP’s graph is complete, so every node has an edge to each of the other vertices in the graph.


$$C\;\mathrm{is}\;\mathrm{symmetric}\;\mathrm{if}\;(\forall i,j:e_{ij}=e_{ji}),$$

In the context of this paper, *e*_*ij*_ equals the distance between the vertices *i* and *j*. Given a set *V* of cities *n* in a two-dimensional space, the objective is to find the optimal Hamiltonian path that minimizes the total tour length [20]:

$$L(\pi |V)={\left\|v_{\pi (n)}-v_{\pi (1)}\right\|}_{2}+\sum_{i=1}^{n-1}{\left\|v_{\pi (i)}-v_{\pi (i+1)}\right\|}_{2}$$ (0.1)

Where ‖.‖_2_ is ℓ_2_ norm and *π* denote as a tour.

## III. Methods

In this section, we will describe the learning algorithm used to train the proposed model. Furthermore, we describe the statistical model used to test whether our proposed model outperforms the GPN.

### 1. Reinforcement learning (RL)

Reinforcement learning (RL) is the process of learning what to perform to increase the expected numerical reward signal. The agent isn’t instructed which actions to perform but must experiment to determine which acts offer the greatest expected reward. To begin, we will define the notation used to represent the TSP as a reinforcement learning problem. Let *S be* the state space and *A* the action space. Each state *s*_*t*_∈*S* is defined as the set of all previously visited cities. Action *a*_*t*_∈*A* is defined as the following selected city from the group of possible cities; our model is considered a sequential one that, given an instance *a*_*t*_ (selected input city) outputs a probability distribution over the next candidates from the remaining cities that have not been chosen. We can define our policy as:

$$P_{\theta }(\pi |x)=P[A_{t}=a|S_{t}=s],$$ (0.2)

From which we can sample to obtain a tour π. In order to train our model, we define the loss [15]:

$$L(\theta |s)=E_{P_{\theta }(\pi |x)}[L(\pi )],$$ (0.3)

Where *L*(*π*) is the cost of the tour that we are attempting to minimize. Recall the REINFORCE’s [22] equation with baseline which is an extension from policy gradient algorithm [23]:

∇J≈E[(L(π)−b(s))∇logπ(a|s)]. (0.4)

Where *b(s)* is the baseline subtracted from the cost to eliminate the policy gradient variance. The optimal baseline is one that lowers variation as much as possible while simultaneously speeding up the training process. As a result, we employ the approach given by [15]:

**Algorithm 1. REINFORCE with Rollout Baseline [** 15 **]**

1: input: number of epochs E, steps per epoch T, batch size B, significance α

2: init θ, *θ*^*BL*^ ← θ # initialize network parameters

3: for epoch = 1,…, E do

4: for step = 1,…, T do

5: *s*_*i*_ ← RandomInstance () ∀ i ∈ {1,…, B} # Generate Random Instances

6: *π*_*i*_← SampleRollout (*s*_*i*_,*p*_*θ*_) ∀ i ∈ {1,…, B} # Sample from policy

7: $\pi_{i}^{BL}$ ← GreedyRollout (*s*_*i*_, $p_{\theta }^{BL}$) ∀ i ∈ {1,…, B} # Greedy selection from policy

8: $\nabla L\leftarrow \sum_{i=1}^{B}L(\pi_{i})-L(\pi_{i}^{BL})\nabla_{\theta }\;log\;p_{\theta }(\pi_{i})$ # Loss calulation((Sampling cost–BL cost)* logprob)

9: θ ← Adam (θ, ∇L) # Optimizer step

10: end for

11: if OneSidedPairedTTest (*p*_*θ*_, $p_{\theta }^{BL}$) < α then # Check if Actor is better than critic with margin α

12: *θ*^*BL*^ ← θ # Transfer Actor’s weights into Critic

13: end if

14: end for

### 2. Linear mixed effect models

Linear mixed effect models (LMEs) are tools used to test whether a significant relationship exists between the dependent variable (response) and independent variables (regressors). LMEs are developed to enable the analysis of dependent data by introducing random variables (i.e., random effects) at the lower levels of the model. For example [24], different algorithms returned solutions to the same instance. To capture the correlation between the solutions of the same instance, a random effect is introduced at the instance level. The formula of the mixed model is shown below

y=Xβ+Zu+ε

Where

*y* is the response vector

*X* is the design matrix for fixed factors

*β* is the coefficients of fixed effect regression

*Z* is the random effects design matrix for random factors

*U* is the vector of random effects

*ε* is the residuals

In the linear mixed model, the fixed factor *x*_*i*_’s null hypothesis is that *x*_*i*_ does not significantly explain some of the variability of the response *y* (i.e., *β*_*i*_ = 0). For the purposes of this paper, the significance level, α, is set at 0.05. To determine if a particular fixed factor significantly affects the response, the p-value corresponding to this fixed factor in the estimation table is compared with the 0.05 significance level.

## IV. Hybrid pointer network (HPN)

HPN is inspired by the Graph pointer network (GPN). GPN is a modified variant of the classic pointer network (PN).

Graph pointer networks have been used to tackle TSP. Building on this approach, in this paper:

- The graph embedding layer is combined with the transformer’s encoder to produce multiple embeddings for the feature context.
- An extra decoder layer is added to operate as a multi-decoder structure network to improve the agent’s decision-making process throughout the learning phase.
- Finally, we switch our learning algorithm from a central self-critic [25] to an actor-critic one as suggested by Kool [15].

**Fig 1:**
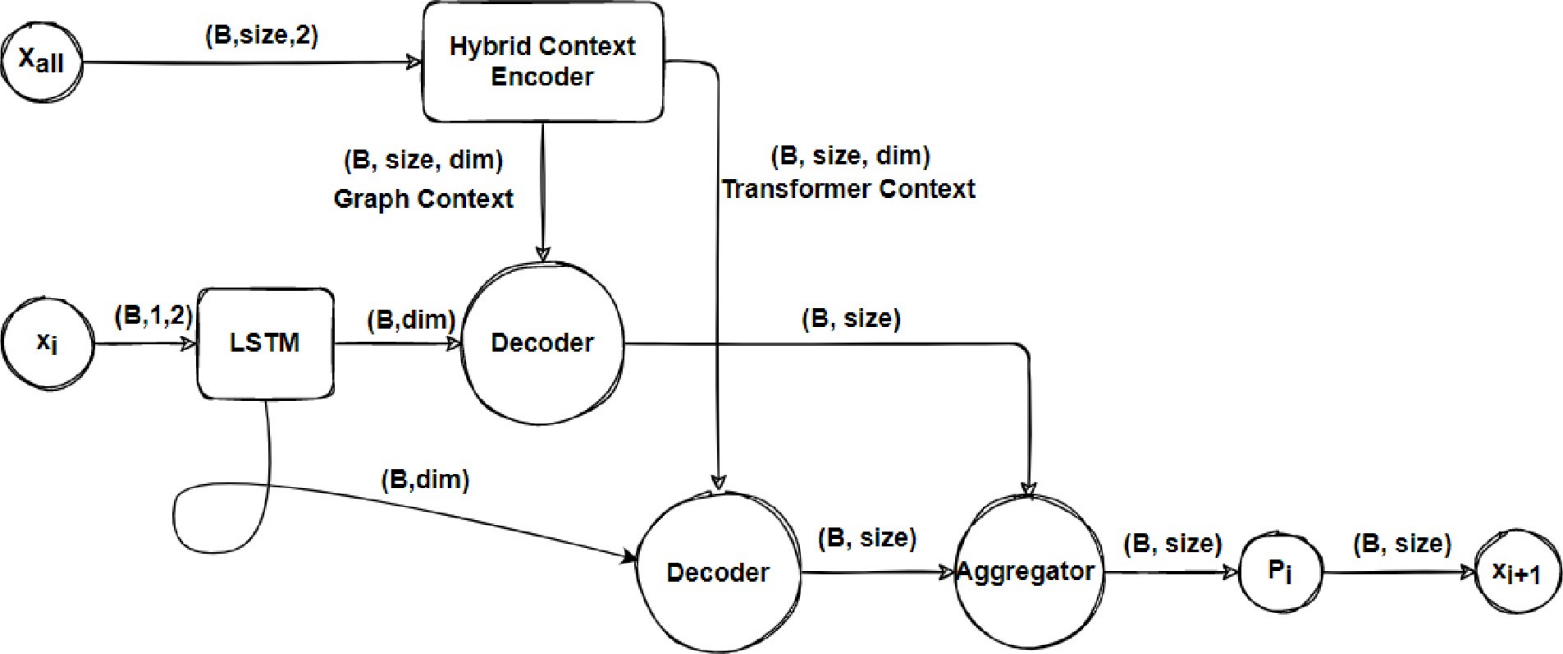
Architecture of HPN which combining a hybrid context encoder with a multi-attention decoder.

The GPN adds graph embedding layer above the pointer network, allowing the model to figure out the complicated relationships between graph nodes in large-scale problems. However, it still struggles to find a globally optimal strategy for TSP problems. This study proposes extending the network architecture to converge to a better policy for small, medium, and large sizes. The proposed HPN is shown in Fig 1. HPN consists of a mixture of several encoder’s architecture and multi decoder based on the attention concept.

### 1. Hybrid encoder

**Fig 2:**
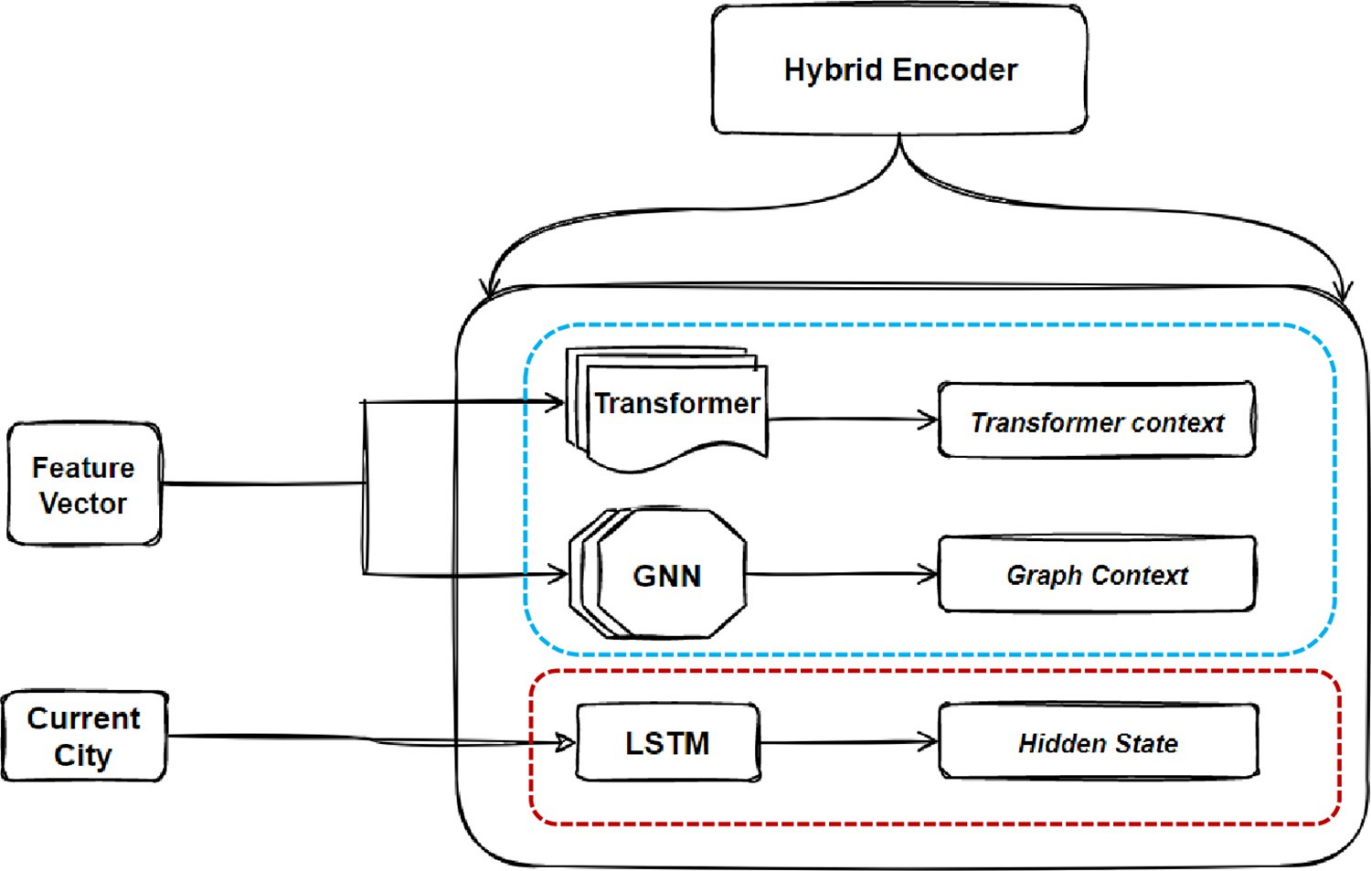
Hybrid encoder consists of transformer’s encoder and graph embedding layer as a hybrid context encoder, (blue dotted box) for the hybrid context encoder and (red dotted box) for the point encoder.

As illustrated in Fig 2, the proposed encoder consists of two parts: the hybrid context encoder, which encodes the Feature vector into two contextual vectors and the point encoder, which encodes the currently selected city by LSTM. Two different encoders are employed for the hybrid context encoder. The first encoder is a typical transformer encoder with multi-head attention and residual connection with batch normalizing layer, the transformer’s encoder equations with a single head are [26]:

$$H^{enc}=H^{l=L^{enc}}\in R^{(n+1)\times d}$$ (0.5)

$$H^{l}=softmax(\frac{Q^{l}K^{l^{T}}}{\sqrt{d}})V^{l}\in R^{(n+1)\times d},$$ (0.6)

$$Q^{l}=H^{l}\;W_{Q}^{L}\in R^{(n+1)\times d},W_{Q}^{l}\in R^{d\times d},$$ (0.7)

$$K^{l}=H^{l}\;W_{K}^{L}\in R^{(n+1)\times d},W_{K}^{l}\in R^{d\times d},$$ (0.8)

$$V^{l}=H^{l}\;W_{V}^{L}\in R^{(n+1)\times d},W_{V}^{l}\in R^{d\times d}$$ (0.9)

Where $W_{Q}^{L}$, $W_{K}^{L}$ and $W_{V}^{L}$ are learnable parameters, *H*^*enc*^ is a matrix contains the encoded nodes, *Q*^*l*^, *K*^*l*^ and *V*^*l*^ are a query, key and value of the self-attention.

The second one is the graph embedding layer. The graph embedding layer context is acquired by directly encoding the context vector obtained from coordinates of cities. Because we are only considering symmetric TSP, the graph is full. As a result, the graph embedding layer can be written as [17]:

$$X^{l}=\gamma \;X^{l-1}\;W_{g}+\left(1-\gamma \right)\varphi_{\theta }(\frac{X^{l-1}}{\left|N(i)\right|})$$ (0.10)

Where $X^{l}\in R^{N\times d_{l}},\;and\;\varphi_{\theta }:R^{N\times d_{l-1}}\to R^{N\times d_{l}}$ is the aggregation function, *γ* is a trainable parameter, $W_{g}\in R^{d_{l-1}\times d_{l}}$ is trainable weight matrix and *N(i)* the adjacency set of node i.

For the point encoder which encodes the currently selected city, each city coordinates *x*_*i*_ (i.e. (*x*_*i*1_, *x*_*i*2_)) is embedded into a higher dimensional vector $\hat{x}\in R^{d}$, where d is the hidden dimension. An LSTM then encodes the vector $\hat{x}$ for the current city *x*_*i*_. The hidden variable $x_{i}^{h}$ of the LSTM is passed to both the decoder of the current stamp and the encoder of the next time stamp.

### 2. Multi-decoder

To begin the decoding phase, a placeholder is added for the first iteration of the decoding to select the best location to start the tour, the decoder is based on the attention mechanism of a pointer network and outputs the pointer vector u_I_, which is then sent through a Softmax layer to build a distribution across the following candidate cities. The attention mechanism and the pointer vector u_I_ are defined as follows [17]:

$$u_{i}^{\left(j\right)}=\{\begin{array}{c} V^{T}.\tanh(W_{r}r_{j}+W_{q}q)\;if\;j\neq \sigma (k),\forall k<j,\\ -\infty \;otherwise, \end{array}$$

Where *u*_*i*_^(*j*)^ is the j-th entry of the vector *u*_*i*_, *W*_*r*_ *and W*_*q*_ are trainable parameters, q is the query vector from the hidden state of the LSTM, is a reference vector containing the contextual information from all cities.

The encoded context from the transformer’s encoder is used as a reference for the first decoder layer and the context obtained from the graph embedding layer is used as the reference for the second decoder layer, as illustrated in Fig 1.

Then we’ll have an attention vector for each decoder layer, and we’ll need to figure out how to aggregate them. For the aggregator Function, four distinct procedures may be used to determine the distribution policy throughout the candidate cities:

- The first option is to add the two attention vectors from each decoder layer, which are provided by:

$$\pi_{\theta }(a_{i}|s_{i})=p_{i}=softmax(u_{i}^{1}+u_{i}^{2})$$

- The second option is to take the maximum value between these two vectors, which is indicated as follows:

$$\pi_{\theta }(a_{i}|s_{i})=p_{i}=softmax(\max(u_{i}^{1},u_{i}^{2}))$$

- The third option is to take the mean as follows:

$$\pi_{\theta }(a_{i}|s_{i})=p_{i}=softmax(average(u_{i}^{1},u_{i}^{2}))$$

- The final option is to concatenate both of them and feed the concatenated vector into a single embedding layer, letting the model to decide how to aggregate them; we can describe this notion as follows:

$$\pi_{\theta }(a_{i}|s_{i})=p_{i}=softmax(\vartheta_{\theta }(cat(u_{i}^{1},u_{i}^{2}))).$$

Where *ϑ*_*θ*_:*R*^*N*×2^→*R*^*N*×1^ is the aggregation function. In the result section, we displayed the outcome for each one of them.

Fig 1 illustrates the model operations. We feed the network a tensor of input nodes in this problem the input nodes contain four features as previously illustrated so the input dimeson will be (batch-size, problem-size, number-of-feature), we feed these nodes into the hybrid context and will get two contextual vectors one from the transformer’s encoder and the other from the graph encoder, then for the first decoding stamp we feed the placeholder to the pointer encoder for learning the best possible location. Finally, we feed the contextual vectors with the hidden states from the pointer encoder to our decoder, which is a simple attention layer, and aggerate the two-attention vectors using the sum operation. For clarity, we employ two decoder layers, one for the context vector of the graph and the other for the context vector of the transformer.

## V. Experiments

In our experiments, the city/node coordinates are independently and randomly drawn from a uniform distribution *x* ~ U(0, 1). In each epoch, the training data is generated on the fly. The hyperparameters provided in Table 1 are used in the following experiments.

**Table 1:** Hyperparameters used for training.

*Parameter*	*Value*	*Parameter*	*Value*
*Graph Embedding Layer*	*3*	*Learning rate*	*1e-4*
*Transformer Encoder Layer*	*6*	*Batch size*	*512*
*Feed-forward dim*	*512*	*Training steps*	*2500*
*Optimizer*	*Adam*	*Tanh clipping*	*10*

### 1. Small-scale experiments

**Fig 3:**
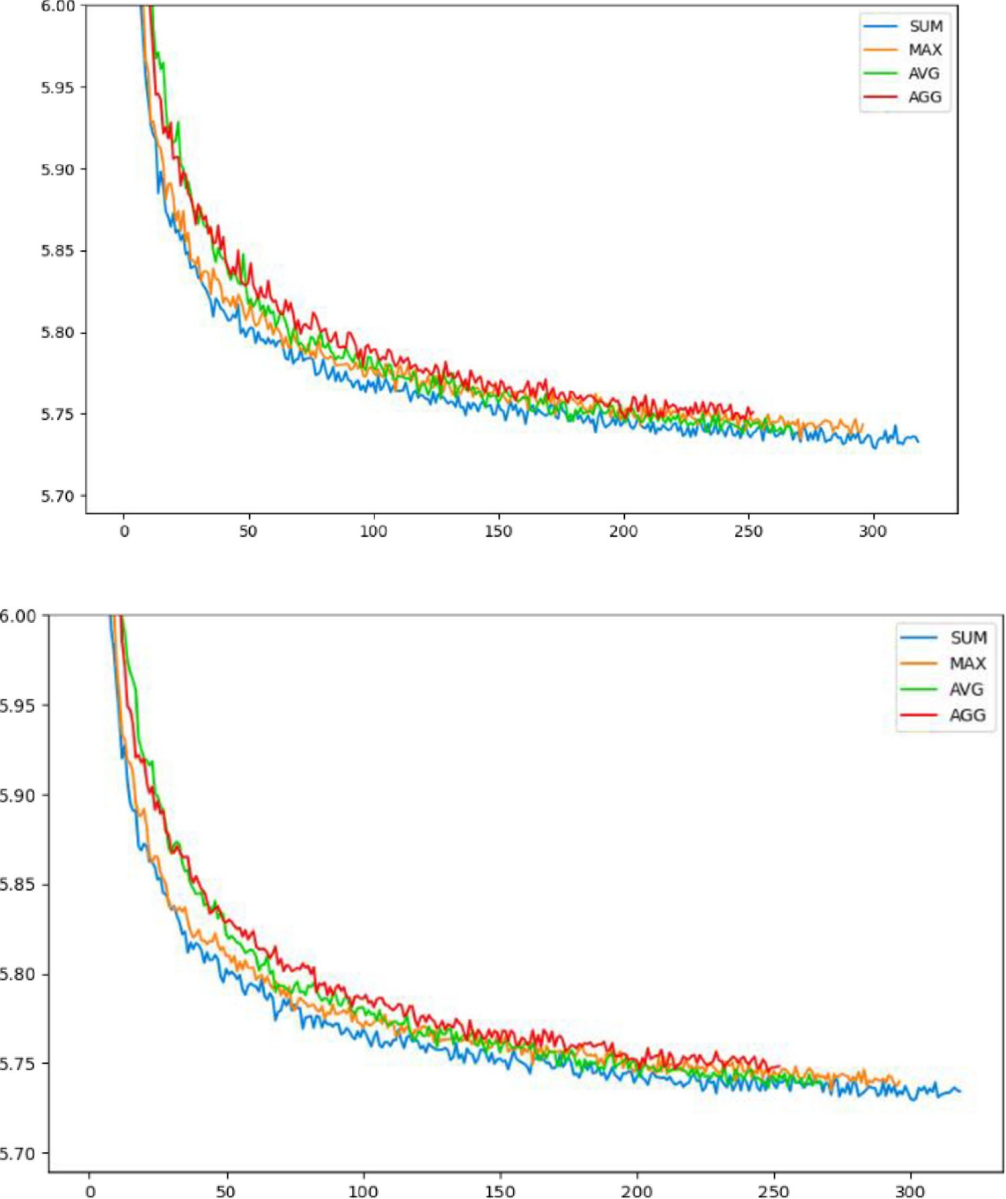
Training performance for the actor (on Top) and the critic (on Bottom) where the total tour length on the y-axis and the number of epochs on x-axis indicating that when we apply the sum operation between the two attention’s vectors, the model converges a little fast compared with the others.

We begin our experiments with a difficult barrier: which aggregator function between the previously described ones will assist our model in achieving better results? Indeed, it is difficult to answer this question without experimenting all of them; we examined the above-mentioned suggestions for this component and recorded the training performance results. Fig 3 illustrate these results.

We can conclude from the above figure that the summation has excellent performance at first, but by the middle of training, the average has caught it, the maximum and the single-layer aggregation have a little higher result, so we decide to stop examining them.

**Fig 4:**
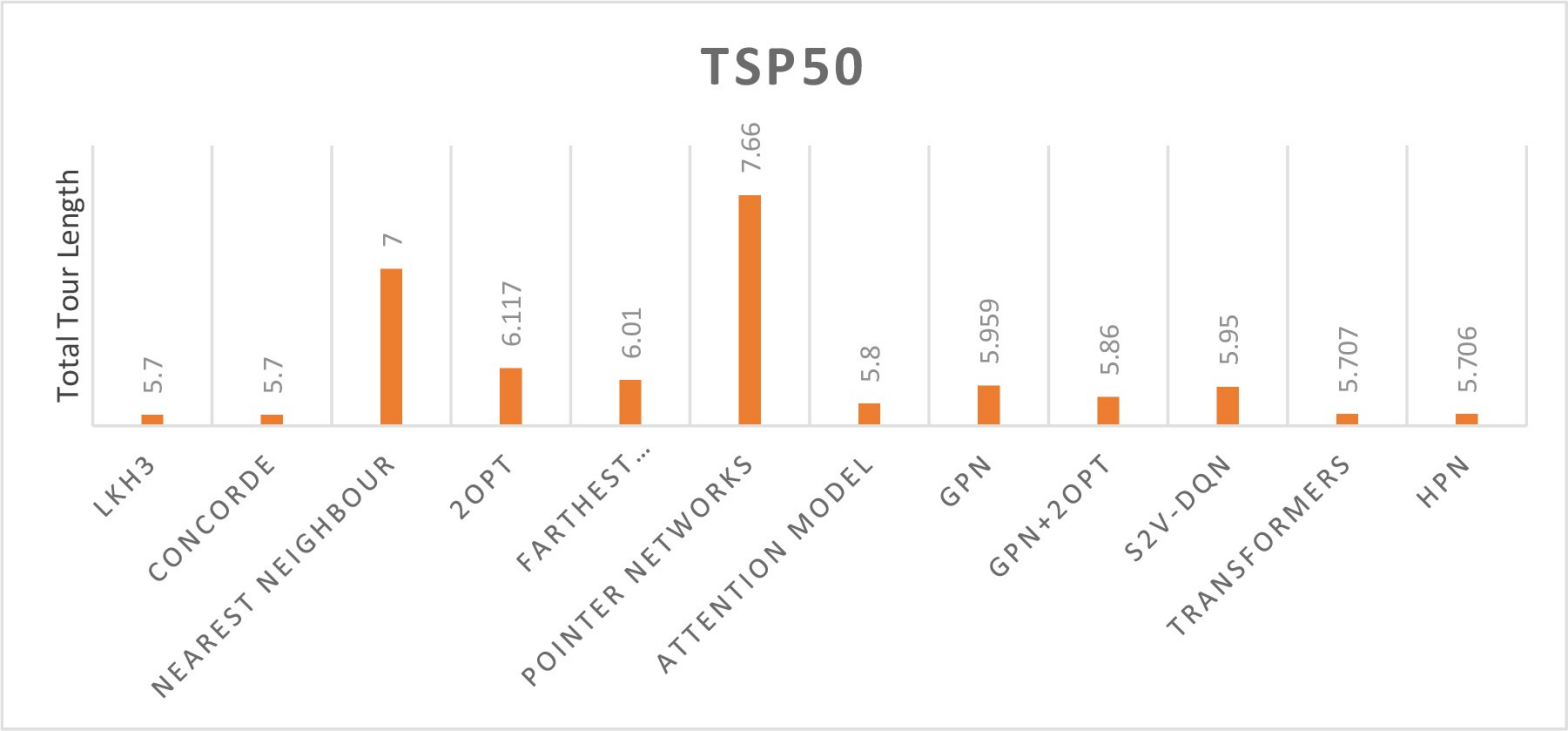
Comparison of TSP50 results.

We use 50 nodes travelling salesman problem (TSP50) instances to train our HPN model to tackle the small-scale. TSP50’s average training time for each epoch is 19 minutes while utilizing one NVIDIA Tesla P100 GPU instance. We compare the performance of our model on small-scale TSP to earlier studies such as Graph pointer networks, the Attention Model, the pointer network, s2v-DQN [3], the Transformer Network [26] and other heuristics, e.g. 2-opt heuristics, Christofides algorithm and random insertion. The results are shown in Fig 4 which compares the approximate tour length to the optimal solution on 10k instances. A small number indicates a better result.

As demonstrated in Fig 4 the optimal solution obtained from CONCORDE, LKH-3 heuristic is an extension of LKH-2 for solving constrained traveling salesman and vehicle routing problems, our HPN model surpasses the current existing models and achieves the state-of-the-art solution for TSP50. Our model outperforms the graph pointer network by a wide margin, enhancing its performance for TSP50 from 5.959 to 5.706 without utilizing 2-opt, which is a success for the hybridization concept.

### 2. Large-scale experiments

For achieving the best possible generalization out of our model, instead of just using the cities’ coordinates as a context for both the graph encoder and the transform encoder, we replace it with a feature context that accelerates the training convergence for the large-scale problems. The feature context includes the vector context previously used by [17] concatenated with the Euclidean distance, where the vector context is just a subtraction operation between the coordinates of the currently selected city with the others.

**Fig 5:**
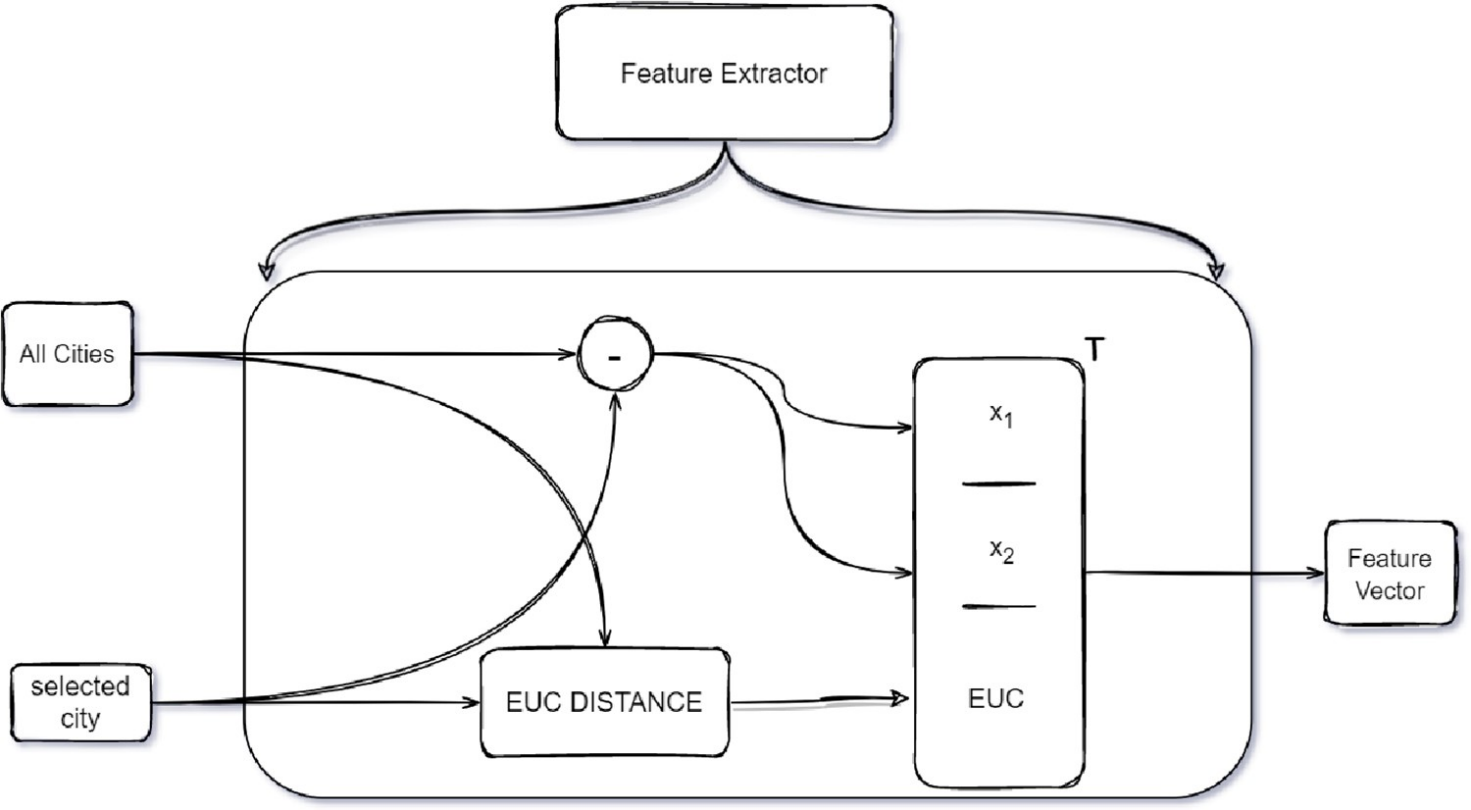
The feature extractor architecture combines both vector context with the Euclidian distance and outputs a Feature vector.

Our feature extractor component does this job as illustrated in Fig 5. It is essential in the proposed HPN model since, it extracts the most relative information and feeds it to the encoder as a context. “Suppose that $X_{i}=[x_{i}^{T},\ldots ,x_{i}^{T}{]}^{T}\in R^{Nx2}$ is a matrix with identical *N* rows. We define ${\bar{X}}_{t}=X-X_{i}$ as the vector context. The j-th row of *X*_*i*_ is a vector pointing from node *i* to node *j* and X is the matrix that contains coordinates of all cities. We expanded this notion by adding the Euclidean distance between the currently selected city and other cities to the vector context.

The Euclidean distance between two points (*x*_*i*1_, *x*_*i*2_) *and* (*x*_*j*1_, *x*_*j*2_) is shown in (0.11):

$$d=\sqrt{(x_{i1}-x_{j1}{)}^{2}+(x_{i2}-x_{j2}{)}^{2}}$$ (0.11)

Where *x*_*i*1_, *x*_*i*2_, *x*_*j*1_ and *x*_*j*2_are the coordinates of city *i* and city *j* respectively.

**Fig 6:**
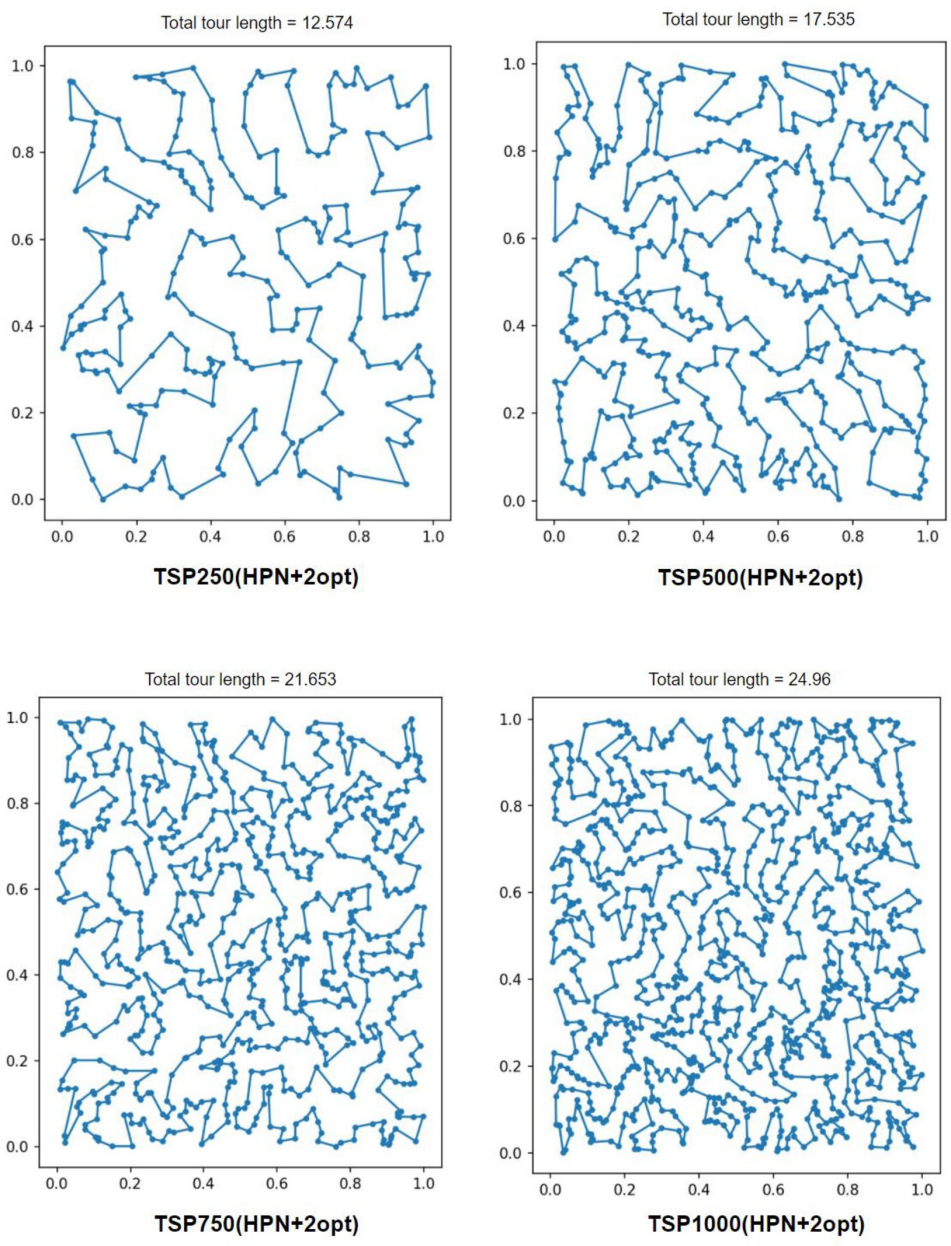
Sample tours for TSP250-500-750-1000 solved by HPN+2-opt.

**Fig 7:**
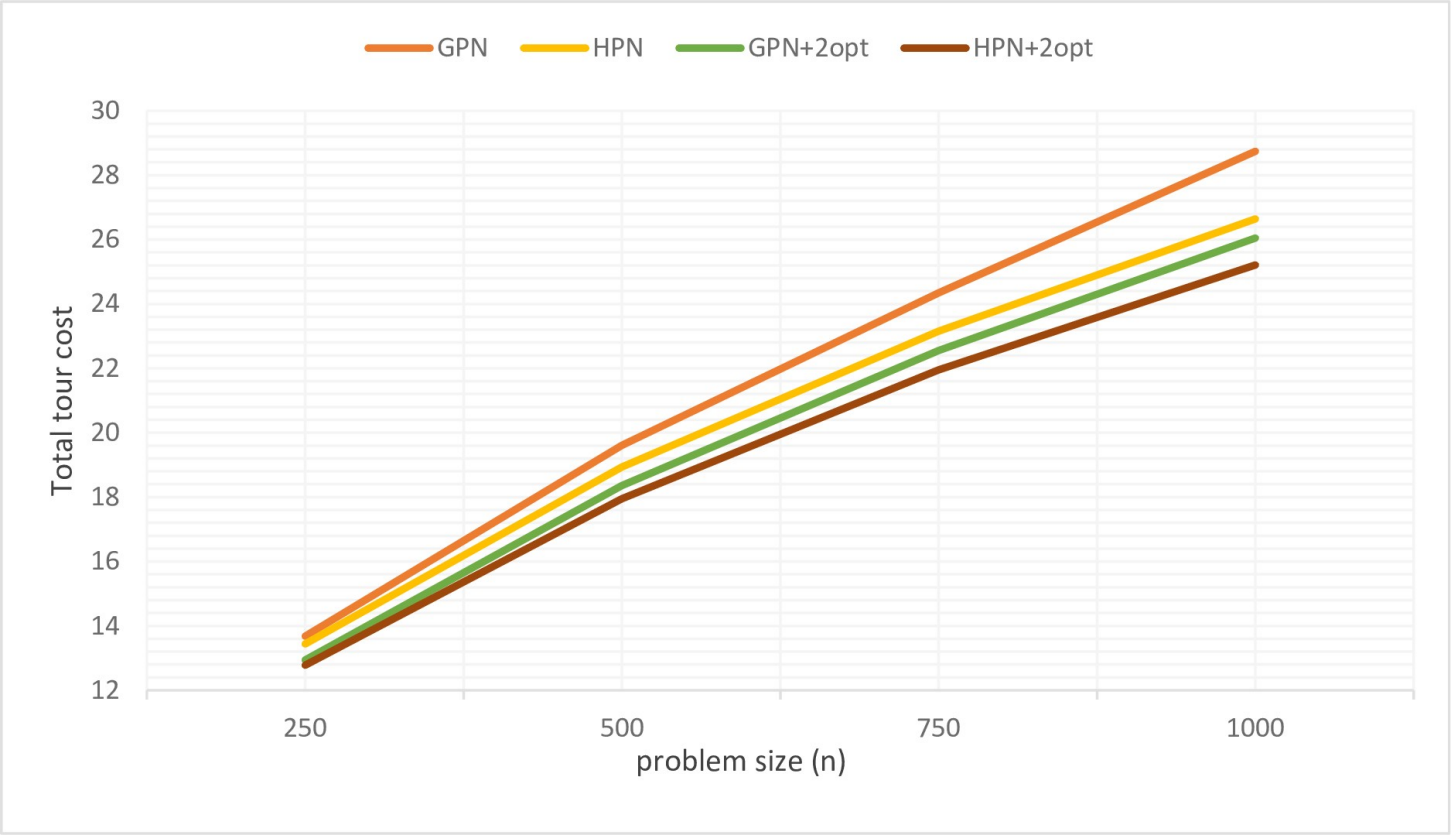
Large-scale results from GPN, HPN, GPN+2opt, and HPN+2opt demonstrate that the gap between our model and GPN increases as the problem size increases.

Using the feature extractor, we train our large model in TSP50, validate with TSP500, 10 epochs, 1e-3 learning rate with leaning rate decay 0.96 and 100 for tanh clipping. Due to memory constraints we only solve 1k instances, some sample tours are shown in Fig 6, in which we solve TSP50-250-500-1000 with HPN+2opt. Table 2 summarizes our result, which shows that our model generalizes better than GPN. For the sake of a fair comparison with the state-of-the-art (i.e., GPN), we used 2opt local search technique to fine-tune the HPN’s tours. As shown in Table 2 and Fig 7, our models outperform the GPN, GPN+2opt, PN, AM, and 2opt models. Moreover, HPN+2opt returns near-optimal tours and generalizes better than the GPN on a large-scale instance.

**Table 2:** TSP’s result using hybrid pointer network model (HPN) vs baselines. Each result is obtained by averaging on 1000 random TSP instances for larger instance and 10k instances for TSP50. Obj is the total tour length and the time reported is for solving 1k instances for larger TSP instances and 10k for TSP50.

Method	TSP50		TSP250		TSP500		TSP750		TSP1000	
	Obj.	Time	Obj.	Time	Obj.	Time	Obj.	Time	Obj.	Time
LKH3	5.70	300s	11.893	9792s	16.542	23070s	20.129	36840s	23.130	50680s
Concorde	5.70	120s	11.89	1894s	16:55	13902s	20.10	32993s	23.11	47804s
Nearest Neighbor	7.00	0s	14.928	25s	20.791	60s	25.219	115s	28.973	136s
2-opt	6.117	7.92s	13.253	303s	18.600	1363s	22.668	3296s	26.111	6153s
Farthest Insertion	6.01	2s	13.026	33s	18.288	160s	22.342	454s	25.741	945s
OR-Tools (Savings)	--	--	12.652	5000s	17.653	5000s	22.933	5000s	28.332	5000s
OR-Tools (Christofides)	--	--	12.289	5000s	17.449	5000s	22.395	5000s	26.477	5000s
Pointer Net	7.66	--	14.249	29s	21.409	280s	27.382	782s	32.714	3133s
Attention Model	5.80	2s	14.032	2s	24.789	14s	28.281	42s	34.055	136s
GPN	5.959	1.75s	13.679	32s	19.605	111s	24.337	232s	28.471	393s
GPN+2opt	5.867	6.5s	12.942	214s	18.358	974s	22.541	2278s	26.129	4410s
s2v-DQN	5.95	--	13.079	476s	18.428	1508s	22.550	3182s	26.046	5600s
Transformers (Gr.)	5.707	13.7s	14.60	4s	23.63	10s	30.77	15s	--	--
HPN (Gr.) **ours**	5.706	0.36s	13.44	16s	18.94	48s	23.15	100s	26.64	168s
HPN+2opt **ours**	--	--	12.78	315s	17.95	1460s	21.95	3405s	25.21	6480s

### 3. Benchmark instances results and statistical analysis

To validate our model against the standard benchmark instances, we employed varied-size instances from the public libraries TSPLIB [27] and World TSP. The benchmark dataset consists of 34 instances. The naming convention of instances consists of the first few letters of the instance location and the problem size n. For example, the instance eg7146 has 7146 points in Egypt. The instance sizes vary from 400 to 10639 nodes(cites). The normalized and actual tour length in km and the testing time in seconds are reported in Table 3.

**Table 3:** Evaluation on real world TSPLIB dataset using HPN and HPN+2opt.

Benchmark	HPN		HPN+2opt		Un-normalized tour length HPN+2opt in km	GPN		GPN+2opt		Un-normalized tour length GPN +2opt in km
	Obj.	Time	Obj.	Time		Obj.	Time	Obj.	Time	
rd400	17.0	2s	**16.6**	2.3s	**16538**	18	1s	16.9	2.1s	16827
gr431	10.4	1.3s	**9.9**	1.8s	**2473**	11.3	1s	10.3	2.3s	2689
d493	13.4	1.3s	**11.2**	3.6s	**38404**	12.9	1.1s	11.7	2.3s	40098
att532	14.8	1.4s	**13.2**	3.4s	**96916**	14.9	1.1s	13.5	3.4s	1029393
pa561	18.0	2.8s	**17.3**	5.3s	**16357**	19	1.3s	17.9	2.8s	16971
u574	18.3	1.4s	**17.2**	3.6s	**41323**	22.7	1.6s	17.5	3.4s	42874
d657	16.3	1.8s	**15.2**	5s	**53921**	17.8	1.6s	15.8	4.1s	55692
gr666	14.5	1.7s	**13.5**	5.3s	**3731**	15.9	1.67s	13.9	5.5s	3840
u724	22.5	2.8s	**21.1**	5.4s	**48742**	22.7	1.5s	21.4	4.7s	49105
rat783	25.1	2.2s	**24.7**	4.4s	**10887**	26.9	1.9s	25	5.1s	11015
dsj1000	18.8	3s	**17.2**	12s	**20715222**	20.1	2.1s	17.5	11.6s	21020136
u1060	23	2.8s	**20**	11.4s	**286109**	23.6	2.3s	21	11.1s	289678
d1291	19.6	4s	**16.6**	17s	**57713**	20.1	2.6s	16.7	20.1s	57964
nrw1379	27.2	4s	**25.8**	15s	**61163**	30.4	3.1s	27.1	19.8s	64005
u1432	33.9	3.8s	**32.8**	14.53s	**166739**	36.2	2.9s	33.6	16.3s	170875
vm1748	28.8	5s	**24.9**	38s	**386483**	29.3	3.8s	25.9	31.15s	395392
rl1889	27	6s	**23.2**	39s	**365362**	29.6	4s	23.6	34.1s	373652
u2152	36.9	6s	**32.3**	43s	**75754**	38.7	4.4s	32.7	42.5s	77622
pr2392	38	7s	**35.2**	70s	**426199**	42.8	5.3s	36.2	57.4s	436979
pcb3038	46.1	10s	**43.**	117s	**154445**	52.7	6.5s	44	105s	156771
nu3496	26.4	9s	**23.8**	144s	**105314**	32.6	8.1s	24.3	154s	107965
fl3795	16.4	2s	**14.7**	170s	**30786**	23.7	8.4s	14.5	258.7s	30402
fnl4461	29.9	15s	**46.7**	220s	**203653**	59	9.3s	49	166.7s	214703
ca4663	26.2	14s	**23.8**	248s	**1585498**	37.2	10.1s	24.3	273.7s	1621202
rl5934	43.9	22.6s	**37.9**	501s	**630300**	54.8	13s	39.6	426.3s	645335
tz6117	46	16s	**40.3**	431s	**434403**	55.6	12.7s	41.6	409.1s	448478
eg7146	21.7	19.3s	**19**	440s	**187376**	35.3	16.7s	19.2	633.3s	189608
pla7397	51.8	26s	**42.6**	906s	**25169496**	71.1	16.3s	44.5	598.4s	26287346
ym7663	32.8	21s	**30.3**	594s	**281001**	57.7	16.6s	30.8	792.5s	286558
ei8246	58.3	22s	**45**	585s	**226498**	75.5	18.1s	55.8	737.4s	233699
ar9152	40.9	26s	**36.9**	834s	**972758**	56.5	19.7s	38.2	1009.3s	1002739
ja9847	28.6	28s	**24.4**	1090s	**542887**	41.5	21.4s	24.7	1438.5s	550364
fi10639	56	49s	**51.5**	1206s	**573948**	81.6	24.2s	52.8	1238.6s	5888001

To understand how HPN is performing compared to the GPN (the state-of-the-art network) in terms of the tour cost and testing time shown in Table 3, we did statistical comparison between these two networks. The statistical model should consider the dependency between the observations shown in Table 3.

In other words, we should realize that the tour cost of HPN and GPN for the same instance are correlated. Moreover, the testing times of same instance using the two networks are correlated as well. Therefore, we used LME regression to explain the variability in the tour cost and testing time (i.e. the responses) [24]. The LME regression model explains the variability of the tour cost and testing time as a function of the network used (i.e., HPN and GPN) and the size of the network. We used one indicator variable called “MODEL” to code the GPN and HPN.


$$MODEL=\left\{ \begin{array}{c} 1\;when\;the\;instance\;was\;solved\;using\;GPN\\ 0\;when\;the\;instance\;was\;solved\;using\;HPN \end{array}\right.$$

In Table 4, we compared the tour cost for the HPN and GPN. A significance level of 0.05 was applied on all regression models. The p-value of the MODEL_1 indicator variable (i.e., GPN) is < .0001 and we conclude that the tour cost of the GPN is statistically significantly higher than the tour cost of the HPN. However, as shown in Table 5, the testing time of the HPN is statistically significantly higher than the testing time of the GPN.

**Table 4:** The fixed effects coefficients of the model explaining the tour cost in terms of the HPN/GPN and the problem size.

Name	Estimate	SE	tStat	DF	pValue	Lower	Upper
(Intercept)	16.01394	2.47475	6.470932	65	< .0001	11.07152	20.95636
size	0.003976	0.000526	7.557891	65	< .0001	0.002926	0.005027
MODEL_1	6.485472	1.229939	5.273005	65	< .0001	4.029116	8.941829

**Table 5:** Fixed effects coefficients of the model explaining the testing time in terms of the HPN/GPN and the problem size.

Name	Estimate	SE	tStat	DF	pValue	Lower	Upper
(Intercept)	0.989527	0.649926	1.522523	65	0.132729	-0.30846	2.287518
size	0.002742	0.000118	23.30437	65	< .0001	0.002507	0.002977
MODEL_1	-2.79206	0.735072	-3.79835	65	< .0001	-4.2601	-1.32402

**Fig 8:**
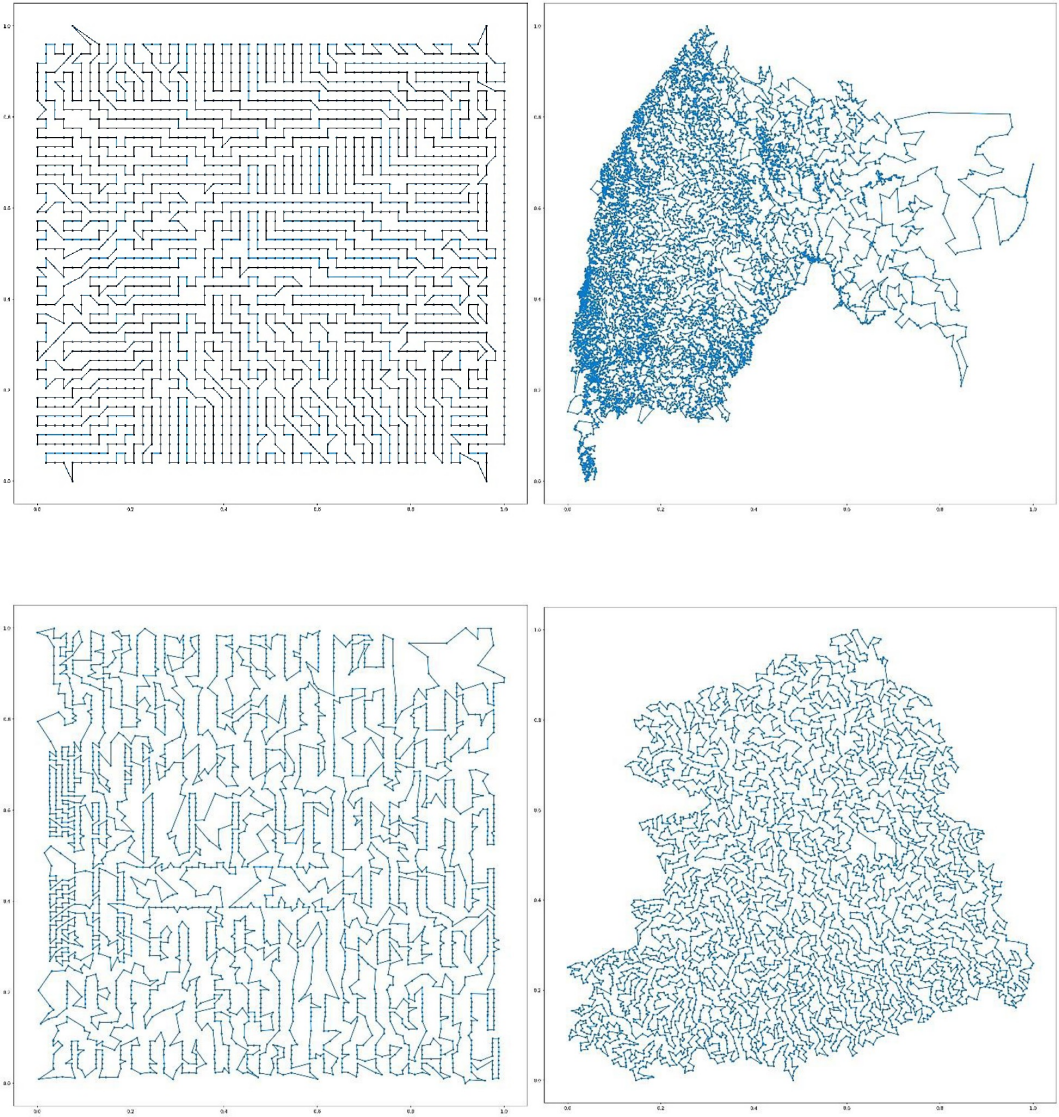
Sample tours for benchmark instances.

We repeat the same analysis for the tour cost and the testing time of the HPN+2opt and GPN+2opt. As shown in Tables 6 and 7, the HPN+2opt has statistically significantly lower testing time and tour cost. This is expected because HPN returns a better tour as an initial point in the solution space which helps 2opt to find a better final tour in less time. Finally, for the sake of completeness, we visualized four constructed tours using HPN + 2opt in Fig 8.

**Table 6:** The fixed effects coefficients of the model explaining the tour cost in terms of the HPN+2opt/GPN+2opt and the problem size.

Name	Estimate	SE	tStat	DF	pValue	Lower	Upper
(Intercept)	17.96973	2.224075	8.079641	65	< .0001	13.52795	22.41152
size	0.002559	0.000488	5.243273	65	< .0001	0.001584	0.003534
MODEL_1	0.724771	0.097485	7.434709	65	< .0001	0.53008	0.919461

**Table 7:** The fixed effects coefficients of the model explaining the testing time in terms of the HPN+2opt/GPN+2opt and the problem size.

Name	Estimate	SE	tStat	DF	pValue	Lower	Upper
(Intercept)	-143.885	25.28218	-5.69116	65	3.26E-07	-194.377	-93.393
size	0.106226	0.005288	20.08878	65	< .0001	0.095665	0.116786
MODEL_1	42.49765	15.33099	2.77201	65	< .0001	11.87955	73.11574

## VI. Conclusion and future work

In this work, a hybrid pointer network (HPN) is proposed for tackling both small-scale and large-scale problems. We demonstrate that the hybrid concept with a graph-based method is successful in improving the model performance for both scales. We used REINFORCE with Rollout Baseline to train our model. Our results show that our model outperforms the traditional graph pointer network with a significant margin, resulting in improved model generalization. Our model is still struggling to find the optimal solution for larger instances. Finding the optimal solution for larger instances is challenging so this will be our future direction.

Our future work, we will attempt to find a robust architecture to improve the quality and the time of solutions for large-scale problems, resulting in better model generalization. We also want to tackle combinatorial problems with constraints, which will be an important direction for future study.
