# Supplementary material for: Hybrid pointer networks for traveling salesman problems optimization
Source: PLoS One. 2021 Dec 14;16(12):e0260995. doi: 10.1371/journal.pone.0260995 (PMC8670669; doi:10.1371/journal.pone.0260995)
Supplement: Attachment — Submitted filename: Reviewer.docx [file pone.0260995.s001.docx]

**Reviewer #1**

1. Please re-write the abstract section. It is recommended that the reference not be in the abstract.

- Thank you! We handled this issue by removing any citations from the abstract.

2. Please write a contribution of this method at the end of the Introduction section.

- Thank you! We added the summary of our contribution at the end of the introduction.

3. Related work is very weak. There are many research in the literature for solving TSP problem by meta-heuristic algorithms. This algorithm has been used a lot and has shown that they can get good performance. Therefore, it is suggested to add 2021 articles in the field of meta-heuristic in the related work section.

- Thank you! We added a more recent method for TSP, and our main concern is in the RL-based models, so we didn't give much attention to meta-heuristic and any method built upon the integer programming, we target the RL-based models so we focused all our effort on that direction.

4. It is recommended to explain the proposed model in a separate section. To be comfortable for readers. (Flowchart is also recommended for the convenience of readers)

- Thank you! That's what we did, we made a separate section (section IV) to illustrate our model and figure 1 illustrate our model's operations graphically.

5. What is the setting of the parameters? ٍ Explain this situation.

- Thank you! It's common hyperparameters used in the literature of RL, and for the sake of fairness, we used the same settings that are used in the paired methods to illustrate that our architecture is the one that improves the performance.

6. The authors should clearly state the limitations of the proposed method in real applications

- Thank you! We added the model's limitation at the end of the conclusion section

7. All the images are blurry, especially the graphs. Provide clearer images.

- Thank you! Handled.

8. This paper has spelling and grammatical errors. Please fix all of them.

- Thank you! Handled

9. Display BKS values in tables.

- Thank you! Our main concern in this study to prove that our model improves the performance of the GPN so we only record the performance for two algorithms to Attract the reader's attention into that point

10. As for the results, they should be commented and should lead to meaningful conclusions. At the moment, the manuscript is simply reporting some numerical values but consideration on the algorithmic behaviour based on the obtained results are not adequately reported.

- Thank you! Handled

**Reviewer #2**

1. the abstract section I too long. Please delete unnecessary sentences.

- Thank you! Handled

2. What is your main contribution? Please highlight it in this manuscript.

- Thank you! We added the summary of our contribution at the end of the introduction.

3. How are the optimal parameters selected? It is well known that modelling complex dynamic systems are challenging. See the recent papers: Discrete farmland fertility optimization algorithm with metropolis acceptance criterion for travelling salesman problems. It can explain the study's method or indicate the contribution in the manuscript.

- Thank you! It's common hyperparameters used in the literature of RL and for the sake of fairness, we used the same settings that are used in the paired methods to illustrate that our architecture is the one that improves the performance.

4. There is an Error in page 11 (Error! Reference source not found), please solve it.

- Thank you! Handled

5. You have used 2opt local search. What is the main reason for choosing this version of local search? If 3opt and LKH are also available.

- Thank you! For the sake of fairness because we compared our model with GPN, and the author used the 2-opt to improve the quality of the solution

6. What is your main purpose in using local search? Generate an iterated LOCAL SEARCH OR multi-start local search model or create a model that simulates both of these mechanisms.

- Thank you! For the sake of fairness because we compared our model with GPN, and the author used the 2-opt to improve the quality of the solution

7. It is recommended to re-examine and design the Figures.

- Thank you! Handled

8. Refer and explain the models used for comparison. For example, LKH-3 is software produced to solve the problems of the TSP and VRP.
   - Thank you! Handled
9. Is Concorde the same as Concorde software? In this case, this software uses algorithms with different settings. Please explain these settings and the algorithm used.
   - Thank you! We took the result as it is from the GPN's paper, so we didn't know the setting used for solving the instances
10. Time criterion is the total execution time or the execution time to get the best solution? Clarify this case, it is vague.
    - Thank you! The time that the model took for solving the 10k instances
11. Please refer to TSPLIB.
    - Thank you! Handled.
12. In evaluations, please use large instances for evaluation.
    - Thank you! Handled.
13. In the results, for example, in the instance (rd400), the value of Obj. shows 17. But in this example, the value of the Best-known solution (BKS) is equal to 15,281. What exactly do you mean by these values?! The results are based on how many independent performances?
    - Thank you! In the first two columns, we report the normalized distance, we downscale the coordinate between 0 and 1 by this way, the model trains fast
14. Please use PDav(%)=( Ave.-BKS)/BKS×100 for better evaluation and comparison.

- Thank you! Our main concern in this study to prove that our model improves the performance of the GPN so we only record the performance for two algorithms to Attract the reader's attention into that point
